# Can Nature Walks With Psychological Tasks Improve Mood, Self-Reported Restoration, and Sustained Attention? Results From Two Experimental Field Studies

**DOI:** 10.3389/fpsyg.2018.02057

**Published:** 2018-10-30

**Authors:** Tytti Pasanen, Katherine Johnson, Kate Lee, Kalevi Korpela

**Affiliations:** ^1^Faculty of Social Sciences/Psychology, University of Tampere, Tampere, Finland; ^2^Melbourne School of Psychological Sciences, University of Melbourne, Parkville, VIC, Australia; ^3^School of Ecosystem and Forest Sciences, University of Melbourne, Parkville, VIC, Australia

**Keywords:** natural environments, restorative environments, green exercise, sustained attention, engagement, psychological well-being

## Abstract

The evidence for restorative effects of contact with nature is vast. Drawing from two well-known theories in Environmental Psychology, Stress reduction theory and Attention restoration theory, restoration can be seen as a sequential, interactive process that begins with physiological relaxation and results in affective and attention restoration and broader life reflection. This interaction between a person and their environment may be facilitated by actively engaging with the environment but this has been understudied. We examined engagement with the environment by asking participants to complete psychological, restoration theory-driven tasks designed to enhance physiological, affective and attention restoration, while walking on nature trails. We conducted two experimental field studies (conceptual replications) in Finland in a coniferous forest (Study 1; *n* = 128) and an urban park (Study 2; *n* = 121). The participants walked at their own pace for 4-6 km with or without psychological tasks. Those in the task conditions completed either theory-based restoration-enhancement tasks or alternative tasks that we expected to be less restorative (Study 1: the same tasks in the reverse order; Study 2: awareness-enhancement tasks). The participants completed self-reports on valence, activation, and restoration, and the Sustained Attention to Response Task, before, and after, the walk. We compared the change between measurements using regression models grouped by study conditions, with age, recent stress, difficulties with wayfinding, start time, and navigation method (Study 2 only) as covariates. Valence and self-reported restoration improved after the walk, but there was no additional benefit from the psychological tasks. In both studies, sustained attention consistently improved following different versions of the restoration-enhancement tasks and, to some extent, after a walk without the tasks. Participants who were more stressed experienced greater improvements in valence and self-reported restoration (Study 1) and sustained attention (Study 2). The results support both Stress reduction theory and Attention restoration theory, and imply that some forms of active engagement with the environment can aid sustained attention but not affective restoration. Future research efforts are needed to replicate these findings and to assess any potential long-term or multiplicative effects of engagement-based tasks, or other strategies that could enhance positive engagement with the environment.

## Introduction

Contact with natural environments has consistently been shown to improve psychological and cognitive outcomes (Hartig et al., 2014). A vast amount of past research has focused on contrasting the effects of urban and natural environments (summarized in a systematic review by Bowler et al., 2010) or on the specific qualities of environments that promote affective or attention restoration (for example, Stigsdotter and Grahn, 2011; Gatersleben and Andrews, 2013). The cognitive processes and the quality of interaction with nature leading to a restorative experience have, however, been underexplored (Markevych et al., 2017) although they are key components in the dominant theories explaining the benefits of contact with nature, Attention restoration theory (Kaplan and Kaplan, 1989) and Stress reduction theory (Ulrich, 1983). In particular, we do not know if the benefits of a nature experience are a result of gaining distance from everyday concerns or if they are rather a result of positive engagement with natural elements (Hartig et al., 2014). Preliminary evidence suggests that focusing on the surrounding environment during nature visits is connected to greater recalled restoration, although it is not the only means of experiencing it (Pasanen et al., 2018). Thus, it may be that active engagement and interaction with the surrounding environment is not a precondition for restorative experiences but it may facilitate them.

Attention restoration theory states that the benefits of interaction with nature are largely due to cognitive benefits and “soft,” effortless fascination (Kaplan and Kaplan, 1989). The theory identifies four qualities that contribute to a restorative experience. *Fascination* implies that there is something in the surroundings that captures one’s attention in a non-depleting, replenishing way (Kaplan and Kaplan, 1989). *Extent* assumes that the environment should have coherent scope such that one feels like being in a whole other world (Kaplan and Kaplan, 1989). *Being away* means being mentally detached from everyday worries and concerns (Kaplan and Kaplan, 1989). Finally, the environment should match one’s current needs to support restoration, thus, *compatibility* is important (Kaplan and Kaplan, 1989). In applied research in environmental psychology, these four qualities have often been interpreted as external, physical qualities, even though Attention restoration theory describes them as components of person-environment interaction (Kaplan, 2001). From this interaction perspective, the role of an individual in need of restoration is an active one, as opposed to being a passive recipient of some pre-determined restorative cues. This idea of active engagement in environmental experiences has been implied in Attention restoration theory, although applied research has not emphasized it (Kaplan, 2001).

Supporting the notion of attention restoration, the cognitive benefits of contact with nature have been demonstrated, from exposure times ranging from 40 s to 55 min (Berto, 2005; Berman et al., 2008; Lee et al., 2015; Pilotti et al., 2015). Recent evidence has suggested that some of the associated cognitive benefits can be enhanced by targeting active engagement with the environment. In a study by Lin et al. (2014), participants were shown five pictures of urban streetscape with trees for a total of 100 s, and their directed attention was measured by the digit span backward task before and after viewing the images. The participants who were instructed to pay special attention to the greenery (trees and plants) in the images improved their directed attention more than another group who were instructed to observe the environment in general (Lin et al., 2014). Thus, focusing specifically on natural features seems to enhance attention restoration.

A similar effect of active engagement on improved cognition has been shown over longer periods in intervention studies (Duvall, 2011, 2013; Lymeus et al., 2018). Lymeus et al. (2018) found improved performance in an attention task followed by 5 weeks of restoration skills training in garden settings, compared with conventional mindfulness training in a classroom with no outdoor views. In Duvall’s studies (Duvall, 2011, 2013), participants were divided into two 2-week walking interventions: a standard condition with planned walking schedules, and an engagement condition where the participants were additionally given several options for engaging with the environment during the planned walks (so called awareness plans). The participants in the engagement group experienced better attentional functioning and less frustration at the end of the study, whereas there was no similar change in the reference group (Duvall, 2011). These results suggest that engagement may be useful for short-term attentional functioning and day-to-day replenishment of cognitive resources.

In the Stress reduction theory (Ulrich, 1983), interaction with the environment is described to start with physiological and initial affective responses, and continue with more elaborated affective, cognitive, and behavioral changes (Ulrich et al., 1991; Hartig et al., 2003). Stress plays a key role in this theory: affective and physiological restoration presumes that the participant is in an initially stressed, highly aroused state that a natural environment helps to restore (Ulrich, 1983). Accordingly, exposure to natural environments have been suggested to function as a buffer that reduces the negative effects of stress on well-being (Wells and Evans, 2003; Mitchell and Popham, 2008; Hartig et al., 2014). Regarding different aspects of stress markers, the evidence is stronger for positive affective changes followed by exposure to natural versus built environments compared with physiological stress indicators (Barton and Pretty, 2010; Bowler et al., 2010; McMahan and Estes, 2015). Thus, it is likely that the physiological effects of exposure to a restorative environment not only appear but also diminish quickly (Hartig et al., 2003).

Potential stress-reducing effects of contact with nature may guide stressed individuals to seek natural environments repeatedly (Russell and Snodgrass, 1987; Gulwadi, 2006). This idea of using and choosing environments for coping is incorporated in the concept of *favorite places* (Korpela, 2003). Favorite places combine the ideas of self- and emotion-regulation, place attachment, place identity, and restoration theories (Korpela, 2012). Most identified favorite places are in natural settings or nearby water, and visits to them provide the more self-reported restoration compared with other types of favorite places (Korpela et al., 2010). However, it is currently not known how common it is to use an environment as a means of stress and emotional regulation (Hartig et al., 2014). Some evidence suggests that adults prefer to go to “classic” natural environment when feeling either happy or sad more than to other types of environments such as urban areas, “unsafe” nature, living rooms, and shopping malls (Johnsen and Rydstedt, 2013).

Even though the restorative experiences described in Stress reduction theory and Attention restoration theory are conceptually different, they have been seen as complementary processes that interact with each other (Kaplan, 1995; Markevych et al., 2017). Stress reduction theory assumes that restoration is a response to visual properties in the environment and their preference evaluation, which quickly results in physiological and affective relaxation (Ulrich, 1983). In Attention restoration theory, the first phase of restoration involves ‘clearing the head,’ that is, removing excessive cognitive residue, followed by recovery of directed attention, facing challenges in one’s mind, and finally, more general life reflection (Kaplan and Kaplan, 1989; Korpela and Hartig, 1996). Integrating these perspectives, Hartig et al. (1991) proposed that a restorative experience begins with physiological and attentional recovery, which are followed by affective changes.

Drawing together Attention restoration theory, Stress reduction theory, and favorite place studies, restoration can be seen as a multi-phasic experience in which individuals can have an active role by interacting with an environment that supports their (restoration) needs. Restorative experiences, in turn, can be important for more general well-being (Hartig et al., 2014). In this paper, we explore whether affective and attention restoration could be enhanced by psychological instructions that aim to deepen the different phases of a restorative experience by conducting two experimental field studies.

## The Present Studies

To study the restorative effects of instructed interaction with the environments, we conducted two field experiments where participants walked along a nature trail, either with or without psychological tasks (descriptives in Table 1). Both studies had two versions of the tasks, one that was hypothesized to be more restorative than the other. The tasks that we hypothesized to be the most restorative were based on restoration theories (Attention restoration theory, Stress reduction theory, and favorite place studies) and their contents followed the different phases of restoration described in the introduction: physiological and affective relaxation, mood-enhancement, building an affective relationship with a place, and general life reflection (Korpela et al., 2017). We labeled these ‘restoration-enhancement tasks.’ The comparison tasks were either the same tasks in the reverse order, that is, mismatched with the hypothesized phases of restoration (Study 1), or ‘awareness-enhancement tasks’ inspired by Duvall’s studies (Duvall, 2011, 2013; Study 2). The participants completed self-evaluated questionnaires on restoration and mood (valence, activation) and a behavioral task on sustained attention before and after the walk.

**Table 1:** Descriptive information of the study settings and the participants.

	Study 1	Study 2
Length (km)	6	4
Environment	Coniferous/mixed forest in the countryside	Urban park near the city center
Where were the tasks read from?	Signposts along the trail	Mobile application
Alternative tasks	Same tasks in the reverse order	Awareness-enhancement tasks (Duvall, 2011)
Design	2 × 2 × 2 (pre-post, tasks/no tasks, route direction)	2 × 3 (pre-post, tasks/no tasks/alternative tasks)
Participants (valid)	150 (127)	122 (119)
Mean age [range]	50 [18–81]	40 [18–63]
Women (%)	80	87


**FIGURE 1:**
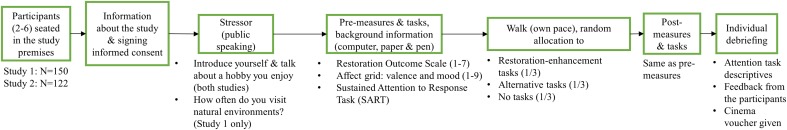
Study procedures.

We hypothesized that walking the nature trails would provide initial benefits: (1a) enhance restoration and valence and reduce activation, and (1b) reduce errors and shorten and stabilize response times in the sustained attention task (Ulrich, 1983; Kaplan and Kaplan, 1989; McMahan and Estes, 2015). We further hypothesized that the above benefits (1a-b) would differ between the study conditions: (2a) the benefits would be greatest after conducting the restoration-enhancement tasks that follow the theory-driven phases of restoration, (2b) the benefits would be smallest after walking without the tasks (due to less interaction with the environment), and (2c) the benefits for those who conduct the comparison tasks would lie between those two. The studies are conceptual replications of each other, with similar procedures (depicted in Figure 1). Study 1 assesses whether any potential restorative effects of conducting the restoration-enhancement tasks depend on the order of the tasks. Is the theory-driven order ideal in terms of experienced restoration after a nature walk? In Study 2, we focus on exploring if the restoration-enhancement tasks have a similar effect as other types of psychological tasks that guide interaction with the environment but do not address restoration in particular. How relevant is the content of the tasks for restorative outcomes? In the next sections, we present the two studies in more detail. At the end of this paper, we return to a more general discussion on the common themes of the studies.

### Study 1 – Coniferous Forest

We began investigating the topic of instructed engagement with the environment during nature visits on a nature trail that had been developed for another project in 2010 (Korpela et al., 2017). For the present study, the trail was equipped with signposts containing the theory-based restoration-enhancement tasks aimed to strengthen affective and attention restoration. We were specifically interested in (1) whether these psychological tasks would aid restoration in general, compared with a walk without tasks, and (2) if the effects of these tasks were stronger when conducted in a theoretically and empirically determined order that mirrored the phases of a restorative experience (physiological, affective, cognitive), compared with the reverse order. Conducting the tasks in the reverse order provided a strong theoretical test, and it was relevant from practical perspective, as the circular route containing the signposts could just as easily be walked in the opposite direction in real life. As the signposts were built into the ground, we assigned four separate groups of participants to walk the route in both directions, with and without the restoration-enhancement tasks.

#### Materials and Methods

##### The study site

The 6-km-long circular trail was located in Ikaalinen, a small municipality in Pirkanmaa, Finland. The before and after measurements were taken at meeting rooms at Ikaalinen Spa, a commercial wellness center that provides both recreational and rehabilitation services. The scenery along the route varied, although it was predominantly a typical Finnish natural environment with lakes, some residential houses, a large sandpit, and forests that were both unpleasant (recently clear-cut forest) and pleasant (a scenic viewpoint by a lake). By the Corine Land Cover 25 ha (2012) classification, approximately 3.2 km of the trail was situated within a ‘coniferous forest,’ 1.2 km (beginning and end around the spa) of the trail were classified as ‘industrial or commercial units’ (with a lake on the side), 1.1 km as ‘mixed forest’ (with the scenic viewpoint), and 0.5 km as ‘fields.’

On average (measured by median and mode), it took 103 min to walk the route, with a range of 65–155 min. The route contained several crossings where the participants were guided by yellow ribbons and printed instructions, containing both pictures and written guidance. Originally, the route with the signposts was marked with arrows that guided visitors to walk in the clockwise direction.

##### Participants

Altogether 150 volunteers participated in 35 sessions (Table 1). Contrary to our initial plan, we could not recruit visitors at the spa and consequently, the majority of participants signed up after reading about the study in a regional newspaper and via the project’s Facebook page. Other recruitment means included a local newspaper, e-mail invitations to local companies, and advertisements at supermarkets in nearby areas. The study was called ‘Forest walk study,’ and the participants were given information about the procedure and the type of measures (e.g., an attention task) but no specific information about the experimental conditions. We conducted one pilot study with volunteer psychology students (*n* = 6) who received no compensation for participation, and a second pilot (*n* = 6), after which the procedure was significantly clarified. Of the remaining 144 participants, a further 15 were excluded due to the following criteria: not walking the instructed route (*n* = 7), problems with the procedure during one study session (*n* = 6), impaired senses (*n* = 1), and personal withdrawal (*n* = 1). For five participants, the attentional task was either not valid or missing. Ten sessions were canceled due to bad weather. The final sample consisted of 129 participants.

For the majority of the sample (92%), the route was new. Many participants showed a special interest in natural environments (we explored this indirectly in the social stressor task, described in Section “Procedure”). In the whole sample, the participants reported visiting nature 3.9 times per week on average, which is more than the national mean of 2-3 times per week (Sievänen and Neuvonen, 2011).

##### Procedure

The procedure is illustrated in Figure 1. The participants came in groups of 2-6 people, mainly from the surrounding municipalities in the region. They were seated in a meeting room in front of a desk with a laptop, a pen, and an envelope that contained the written tasks. First the researchers (most commonly two project workers) introduced themselves, the study, and the procedure, after which the participants signed the informed consent. Further information about the experiment was then detailed. The participants were asked not to talk aloud during the measurements and to refrain from using mobile phones during the study.

We conducted the experiments during the holiday season (May-September 2016) when stress levels may be lower than usual (de Bloom et al., 2010). To induce a mildly stressed state that could potentiate restorative effects (Ulrich, 1983), we started with a social stressor task, after which the participants completed the self-reported questionnaires and the behavioral measurements. When they were finished, the participants left the room in their own pace and they were given verbal and written instructions for the walk one by one outside the study room. The participants were instructed to walk by themselves. Before and after the walk, the participants could help themselves to some fruit, fresh juice, and water. After the walk, the respondents returned to the study room to complete the tasks in the same order as before the walk. At the end of the session, we showed each participant descriptive statistics of their attention task results, asked for feedback on the study, and gave everyone a cinema voucher. The procedure took approximately 2.5-3 h per person.

In addition to the measures reported in this paper, the participants completed self-reported measures of empathic feelings and vitality and a behavioral task of frustration tolerance, but these are reported elsewhere due to space constraints and different theoretical reasoning. The study was carried out in accordance with the recommendations for “Responsible conduct of research and procedures for handling allegations of misconduct in Finland 2012” by the Finnish advisory board on research integrity (TENK). The protocol was approved by the Ethics Committee of the Tampere Region. All subjects gave written informed consent in accordance with the Declaration of Helsinki.

##### Study conditions

To control for any effects of weather, the participants were randomly allocated to different walking conditions each study day: 1/3 were assigned to a walk with the restoration-enhancement tasks completed in the designed, theory-based order (which we will call ‘clockwise order’ because they walked the route in the clockwise [C] direction), 1/3 were assigned to a walk with the restoration-enhancement tasks completed in the reverse order (hence, they walked the route in the reverse [R] direction), and the rest to a walk without tasks, of which one half (1/6 of the sample) walked the clockwise (C) and another half (1/6) the reverse (R) route. The participants in the ‘no task’ conditions walked the route in opposite directions to account for any potential environmental differences, and the initial idea was to combine these conditions for the analyses.

##### The psychological instructions

The instructions on the signposts were based on Stress reduction theory (Ulrich, 1983; Ulrich et al., 1991), Attention restoration theory (Kaplan and Kaplan, 1989), and favorite place studies (Korpela et al., 2008; Korpela and Ylén, 2009). Integrating these theories, a restorative experience has been suggested to start with physiological relaxation, followed by affective and mood-enhancing responses, and advance to building an affective relationship with the place and reflection on one’s current situation in life (Korpela et al., 2017). Thus, the first three signposts related to physical relaxation and observing the environment (for example, “[…] Keep looking around and let yourself be enchanted by your surroundings. Keep breathing peacefully.”), the next two to favorite place identification and reminiscence (“Find your favorite place in this area […] Choose a detail by which you may remember this place, perhaps for years.”), and the final two to clearing the mind and life reflection (“Look around for something representing you or your current situation in life […] Are you gaining new thoughts?”).

##### Pre- and post-walk measures

*Self-reported restoration* was measured with the 6-item Restoration Outcome Scale (ROS; Korpela et al., 2008; see also Hartig et al., 1998; Staats et al., 2003). The scale is a self-evaluation of attention restoration (one item: “I feel alert and able to concentrate”), relaxation and calmness (three items, for example, “I feel restored and relaxed”), and clearing one’s thoughts (two items, for example, “My thoughts are clear”). Participants rated their current state on a 7-point rating scale ranging from “Describes my experience…” 1 = not at all to 7 = completely. We calculated the mean summary score of the responses in both pre- and post-measurements (Cronbach’s α = 0.85 and 0.89, respectively).

*Mood* was measured with a two-dimensional affect grid (Russell et al., 1989) in which the participants are asked to evaluate their mood by marking a single cross in a 9 × 9 grid. The axes reflect core affects, *valence* (horizontal axis) and *activation* (vertical axis; Russell et al., 1989; Västfjäll and Gärling, 2007).

*Sustained attention* was measured using the Random version of the Sustained Attention to Response Task (SART), a test of sustained attention (Robertson et al., 1997). In the SART, participants respond to the digits 1–9, presented in a random order (each shown 25 times in five different font sizes) on a screen for 4.3 min. They were instructed to press the space bar whenever they saw any digit (Go) except the digit 3 (No-Go). The participants were asked to pay equal attention to speed and response accuracy. The stimulus was shown for 250 ms, followed by a mask (a white cross within a circle) for 900 ms. We used the source code provided by Stothart (2015) in the open-source software PsychoPy (Peirce, 2009), in which we translated the instructions into Finnish. The participants were seated approximately 40 cm from the screen of a Dell Latitude laptop, although they were free to move further or closer during the experiment. Both pre- and post-tests were preceded by a practice round with 18 digits where the participants received immediate feedback on the accuracy of the response (correct/incorrect).

The SART provides a number of sustained attention measures. Commission errors – the number of responses made to the No-Go digit ‘3’, reflect response accuracy, controlled attention (Manly et al., 2003), and response inhibition (Johnson et al., 2007). Omission errors - the number of non-responses to a Go digit - had a median of 1 and thus, there was little variation to examine and we excluded the measure from the analyses. The mean and standard deviation (SD) of response time (RT) were calculated after excluding responses to the digit ‘3’ and RTs < 100 ms. SDRT reflects the stability of the response style, with larger variability indicating more attentional lapses (Robertson et al., 1997; Manly et al., 2003; Smilek et al., 2010). The sequence of 225 RTs per participant was further analyzed using a Fast Fourier Transform (FFT) based on the method described in Johnson et al. (2007). Two dependent measures were derived from these FFT analyses – the slow (SFAUS) and fast (FFAUS) frequency areas under the spectra. For the SFAUS, the RT data were analyzed over the entire task. For the FFAUS, the RT data were analyzed in a first half versus second-half analysis. The SFAUS is a measure of all sources of variability in RT slower than 0.0772 Hz, which is derived from the Fixed version of the SART and represents one cycle of a presentation of the digits 1–9 (Johnson et al., 2007), and it measures gradual change in speed of responding over the course of the task. The FFAUS is a measure of all sources of variability faster than 0.0772 Hz, representing trial-to-trial variability in responding, and it measures moment-to-moment variability in responding.

##### Covariates

*Stress* in the past 4 weeks, which potentiates restoration effects (Ulrich, 1983), was measured by 10-item Perceived Stress Scale (Cohen et al., 1983), of which we calculated the summary score (Cronbach’s α = 0.84). *Age* was asked in full years. Older samples have been found to experience greater affective changes after nature exposure (McMahan and Estes, 2015) but we also hypothesized that older participants may find the lengthy route more exhausting, which could be reflected in lower restorative changes. For the majority of participants, the *start time* was at 10.30 am but it varied from 10 am to 4 pm to accommodate as many participants as possible. Time of day can, however, influence the level of alertness and task performance (Monk and Leng, 1982). We coded the start times as -1 = morning (10 - 10.30 am), 0 = midday (12 am - 1 pm), and 1 = afternoon (3 - 4 pm). As a *post hoc* measure, we recorded if the participants reported *problems with wayfinding* during the walk. Having to focus on navigation in a new environment requires mental effort which can reduce any potential restorative effect (both attentional and affective; Gatersleben and Andrews, 2013). We also recorded walk duration, weather, temperature, gender, and the number of hours slept the night before but these were not related to the outcomes in either of the two studies (Appendices A, B, D, E).

##### Data analysis

The *a priori* sample size was calculated as a 3 × 2 between-group repeated measures MANOVA with several correlating dependent variables, with a power of = 0.95 and alpha = 0.05. In this type of design, a medium effect size of 0.25 would be detected with a sample size of 165 participants (Gpower 3.1 software; Faul et al., 2007). However, as the final number of valid cases was lower than we aimed for, the following analyses have less statistical power than we expected to have.

Prior to the actual analysis, we checked that there were no differences between the groups at baseline in any of the outcomes with a one-way analysis of variance (ANOVA) in SPSS version 24 (provided in Appendix C). We also checked for differences in the outcomes between the two ‘no task’ groups that walked the route in different directions. Our initial plan had been to combine these two groups but as there were differences between them, we kept them separate in the analyses. However, we interpreted the results related to them with caution due to their smaller sample size.

We compared the change between pre- and post-measurements with multigroup regression analysis using Mplus version 7.4. The data was continuous but non-normally distributed so the MLR estimator was used (Muthén and Muthén, 1998/2012). The grouping was based on the direction of the route (clockwise/reverse), and completing the restoration-enhancement tasks was an explanatory variable (for simplicity, however, we present these estimates in the results as the difference between within-group intercepts, that is, the estimated within-group means). To retain more power in the analyses, we pre-selected those covariates that correlated significantly (*p* < 0.05) or showed a significant mean difference (in ANOVA) in at least one of the outcomes in either Study 1 or Study 2 (if applicable; these analyses are provided in Appendices A, B, D, E). Continuous covariates were centered and ordinal/dichotomous covariates were recoded so that their midpoint was at 0. In the initial models, the covariates were assumed to have a similar effect in both groups. If the standardized residuals for the covariates were large (>|1.96|), we relaxed this assumption and retained the modified model if the overall model fit improved.

In addition to the residuals, we checked how the models fit with the data and compared the models with the following criteria: a non-significant χ^2^-test, Satorra–Bentler corrected χ^2^ difference-test (for model comparison), smaller values for information criteria (Akaike’s Information Criteria [AIC], Bayesian information criteria [BIC], and sample-adjusted BIC), Comparative Fit Index (CFI) and Tucker-Lewis Fit Index (TLI) ≥ 0.95, the Root Mean Square Error of Approximation (RMSEA) ≤ 0.05, and the Standardized Root Mean Square Residual (SRMR) ≤ 0.08 (Tucker and Lewis, 1973; Bentler, 1990; Browne and Cudeck, 1992; Hu and Bentler, 1999; Satorra and Bentler, 2010; Kline, 2016). To check for influential outliers we examined Cook’s distances in the first models for each block of outcomes, and if they exceeded 1.00 (Tabachnick and Fidell, 2014), the analyses were re-run without the most influential cases by excluding them one by one. If excluding an influential outlier improved the model fit, we retained the improved model.

To account for correlations between related outcomes but to retain more power in the analyses, we analyzed the outcomes in blocks of three: (1) self-reported measures (restoration, valence, and activation); (2) traditional SART measures (commission errors, RT, and SDRT); (3) refined SART variability measures (FFAUS in the 1st and 2nd halves of the tests, and SFAUS).

##### Sensitivity analyses

If applicable, we ran two types of sensitivity analyses for the final models: (1) for those models where we deleted influential outlier(s), we re-ran the final models with those outliers, (2) for the model with refined SART variability measures, we re-ran the models excluding participants whose mean RT was > 500 ms. RTs > 500 ms are generally considered slow in SART studies with adult participants and slower RTs can be connected to inflated FFAUS and SFAUS, which, in turn, may bias the model estimates. We ran these second sensitivity analyses to assess whether the results for FFAUS and SFAUS were influenced by respondents with slow mean RTs.

#### Results

##### Self-reported restoration and mood

**FIGURE 2:**
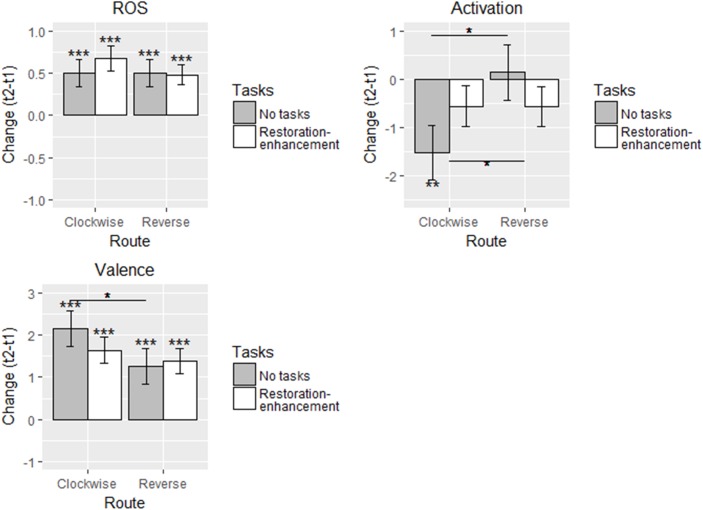
Adjusted means in different conditions for the self-reported measures in Study 1 (*n* = 129). Solid line: statistically significant between-group difference. ^∗^*p* < 0.05, ^∗∗^*p* < 0.01, ^∗∗∗^*p* < 0.001.

Participants in all conditions reported greater restoration after the walk but there were no differences between the conditions (supporting hypothesis 1a but not 2a-c; Figure 2 and Table 2). The estimated change varied, on average, between 0.48 and 0.67 units on the original 1-7 scale. Similarly, in terms of estimated valence, hypothesis 1a but not 2a-c gained support, as the participants reported feeling, on average, 1.27-2.16 units more pleasant after the walk in all conditions. Activation, in turn, did not change in most groups which was against our hypotheses 1a and 2a-c. The exception were the participants in the ‘no task’ (C) condition who felt 1.52 units calmer after the walk.

**Table 2:** The results for multigroup regression models for the self-reported measures in Study 1 (*n* = 129).

		Self-reported restoration				Valence				Activation			
				
		*b*	*SE*	*p*	β (C/R)	*b*	*SE*	*p*	β (C/R)	*b*	*SE*	*p*	β (C/R)
Mean difference, estimated	(1) Restoration-enhancement tasks (C)	0.67***	0.15	0.00	0.84	1.64***	0.30	0.00	0.99	-0.56	0.42	0.19	-0.23
	(2) Restoration-enhancement tasks (R)	0.48***	0.12	0.00	0.66	1.39***	0.30	0.00	0.84	-0.56	0.41	0.17	-0.28
	(3) No tasks (C)	0.50***	0.16	0.00	0.63	2.16***	0.42	0.00	1.30	-1.52**	0.57	0.01	-0.62
	(4) No tasks (R)	0.50***	0.17	0.00	0.69	1.27***	0.42	0.00	0.76	0.14	0.57	0.77	0.07
	Task × route interaction (difference ‘1–3’ – ‘2–4’)	-0.19	0.26	0.47		0.64	0.57	0.26		-1.66*	0.83	0.05	
Covariates	Stress	0.24*	0.11	0.03	0.17/0.19	0.38	0.30	0.21	0.13	-0.29	0.36	0.42	-0.07/-0.08
	Start time	0.03	0.11	0.75	0.03/0.04	0.23	0.23	0.33	0.09/0.11	-0.24	0.32	0.45	-0.07/-0.10
	Age	-0.01	0.00	0.05	-0.15/-0.18	-0.01	0.01	0.57	-0.05	0.00	0.01	1.00	0.00
	Wayfinding problems	-0.70**	0.23	0.00	-0.28/-0.31	-1.25*	0.56	0.02	-0.24	-0.08	0.56	0.88	-0.01
*R*^2^ (C/R)		0.20/0.21				0.11/0.11				0.04/0.04			


*χ^*2*^ = 7.89 (df = 15, *p* = 0.93), RMSEA < 0.001, CFI = 1.00, TLI = 1.28, SMRM = 0.04. Grouping is based on walking direction: C, clockwise; R, reverse route; figures separated by “/” if they differ between the groups. ^∗^*p* < 0.05, ^∗∗^*p* < 0.01, ^∗∗∗^*p* < 0.001.*

The change in restoration was greater for younger and more stressed participants (Table 2). Having a problem with wayfinding was connected to a more negative change in both self-reported restoration and a less positive mood (Table 2). Start time was not connected to changes in the self-reported measures.

The model explained self-reported restoration best (*R*^2^’s 0.20-0.21), followed by valence (0.11) and activation (0.04). The model fit well with the data and no influential outliers were excluded or large residuals freed (Table 2).

##### Sustained attention – traditional measures

**FIGURE 3:**
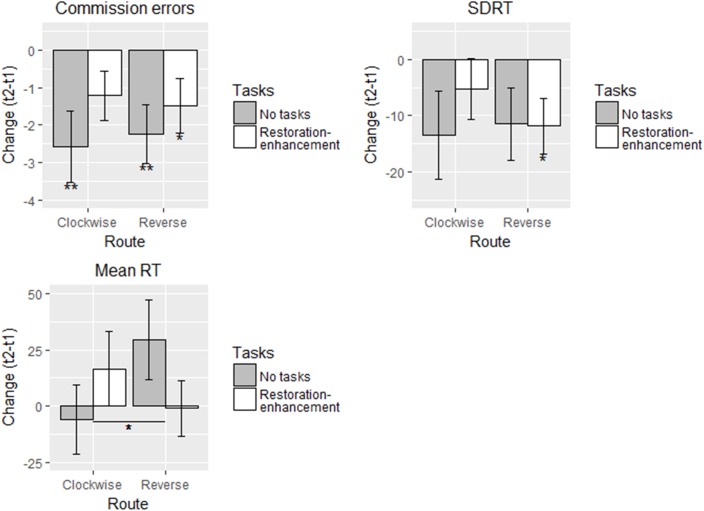
Adjusted means in different conditions for the traditional SART measures in Study 1 (*n* = 125). Solid line: statistically significant between-group difference. ^∗^*p* < 0.05, ^∗∗^*p* < 0.01, ^∗∗∗^*p* < 0.001.

The participants who either walked without tasks or conducted the restoration-enhancement tasks in the reverse order made 1.49 - 2.57 less commission errors after the walk (Figure 3 and Table 3), supporting hypothesis 1b in these groups. The trend was the same for the participants who conducted the restoration-enhancement tasks in the clockwise order, although the estimate (-1.22) was not statistically different from zero (Table 3). Similarly, SDRT reduced significantly in the condition with the reversed restoration-enhancement tasks, and the trend was to the same direction in both ‘no task’ conditions (showing partial support for hypothesis 1b but not 2a-c). With mean RT, there were no significant changes before and after the walk in any of the conditions (contrary to hypothesis 1b) but there was an unexpected interaction effect between route and tasks. Conducting the tasks was associated with increased mean RT compared with not conducting them in the clockwise route, whereas in the reverse route, conducting the tasks was associated with decreased mean RT compared with not conducting them (Figure 3). All these results were in contrast with our hypotheses 2a-c because they indicated the least benefits from conducting the restoration-enhancement tasks in the clockwise order.

**Table 3:** The results for multigroup regression models for the traditional SART measures in Study 1 (*n* = 125).

		Commission errors				Mean RT (ms)				SDRT (ms)			
				
		*b*	*SE*	*p*	β (C/R)	*b*	*SE*	*p*	β (C/R)	*b*	*SE*	*p*	β (C/R)
Mean difference, estimated	(1) Restoration-enhancement tasks (C)	-1.22	0.66	0.07	-0.30	16.64	16.29	0.31	0.20	-5.28	5.46	0.33	-0.17
	(2) Restoration-enhancement tasks (R)	-1.49^∗^	0.73	0.04	-0.37	-0.86	12.35	0.94	-0.01	-11.86^∗^	5.00	0.02	-0.44
	(3) No tasks (C)	-2.57^∗∗^	0.94	0.01	-0.63	-5.83	15.31	0.70	-0.07	-13.44	7.77	0.08	-0.43
	(4) No tasks (R)	-2.24^∗∗^	0.77	0.00	-0.56	29.21	15.31	0.10	0.41	-11.44	7.77	0.08	-0.42
	Task × route interaction (difference ‘1–3’–‘2–4’)	-0.61	1.47	0.68		-52.55^∗^	25.10	0.04		-8.59	10.88	0.43	
Covariates	Stress	-0.92	0.64	0.16	-0.13	30.06/-5.20	19.56/12.44	0.12/0.68	0.20/-0.04	-5.20	4.79	0.28	-0.09/-0.11
	Start time	-0.40	0.47	0.39	-0.07/-0.08	9.17	10.28	0.37	0.07/0.10	-2.91	3.87	0.45	-0.06/-0.09
	Age	-0.02	0.02	0.44	-0.06/-0.07	0.23	0.44	0.61	0.04/0.05	-0.19	0.19	0.32	-0.08/-0.11
	Wayfinding problems	2.91^∗∗^	0.98	0.00	0.23/0.24	-57.44^∗^	22.73	0.01	-0.22/-0.26	-15.15	8.60	0.08	-0.16/-0.18
*R*^2^ (C/R)		0.09/0.09				0.12/0.13				0.05/0.07			


*χ^2^ = 2.87 (df = 13, *p* = 1.00), RMSEA < 0.001, CFI = 1.00, TLI = 1.25, SMRM = 0.02. Grouping is based on walking direction: C, clockwise; R, reverse; figures separated by “/” if they differ between the groups. Parameters freed across groups: RT regressed on stress, covariance between commission errors and SDRT. ^∗^*p* < 0.05, ^∗∗^*p* < 0.01, ^∗∗∗^*p* < 0.001.*

Age, stress in the past week, or start time were not significantly connected to changes in the outcomes but reporting problems with wayfinding was (Table 3). Those who reported problems with wayfinding made almost three more commission errors and had a significantly faster mean RT after the walk (Table 3).

The variances explained were nearly 0.09 for changes in commission errors, 0.12-0.13 for changes in mean RT, and 0.05-0.07 for changes in SDRT. The model with two freed parameters fit the data well (Table 3).

##### Sustained attention – refined variability measures

**FIGURE 4:**
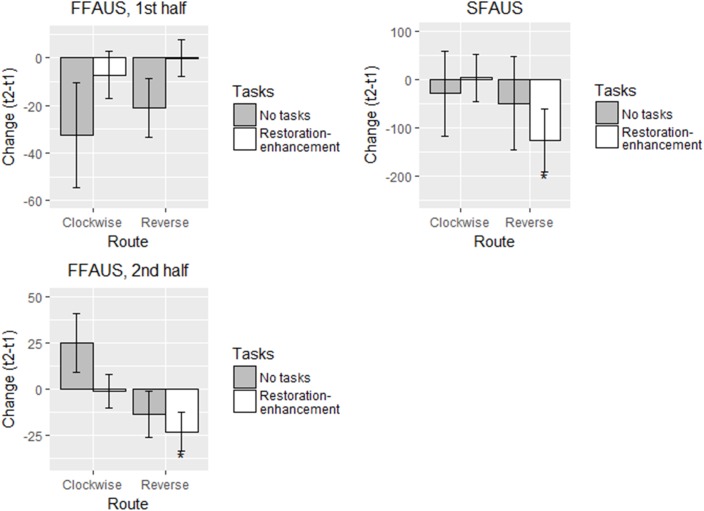
Adjusted means in different conditions for the refined SART variability measures in Study 1 (*n* = 118). Solid line: statistically significant between-group difference. ^∗^*p* < 0.05, ^∗∗^ *p* < 0.01, ^∗∗∗^*p* < 0.001.

In the refined SART variability measures, there were several influential outliers and even after deleting the four most influential ones, the standard errors of the intercepts were large (Figure 4 and Table 4). The participants had similar amounts of FFAUS in the first half of the tasks (against hypotheses 1b and 2a-c), whereas in the second half only the group who conducted the restoration-enhancement tasks in the reverse order showed reduced FFAUS (partially supporting hypothesis 1b; Figure 4 and Table 4). Similarly, this group performed the SART with less SFAUS throughout the whole test after the walk, whereas the other groups showed no change. Our hypothesis 1b was, therefore, supported in only one group, and this group was not the one we hypothesized (2a) to show the greatest improvements.

**Table 4:** The results for multigroup regression models for the refined SART variability measures in Study 1 (*n* = 118).

		FFAUS, 1st half				FFAUS, 2nd half				SFAUS			
				
		*b*	*SE*	*p*	β (C/R)	*b*	*SE*	*p*	β (C/R)	*B*	*SE*	*p*	β (C/R)
Mean difference, estimated	(1) Restoration-enhancement tasks (C)	-7.21	9.98	0.47	-0.09	-1.19	8.96	0.89	-0.02	3.51	48.72	0.94	0.01
	(2) Restoration-enhancement tasks (R)	-0.15	7.52	0.98	0.00	-23.23^∗^	10.57	0.03	-0.42	-125.97^∗^	64.06	0.05	-0.34
	(3) No tasks (C)	-32.48	22.20	0.14	-0.42	24.92	15.86	0.12	0.41	-29.36	87.85	0.74	-0.11
	(4) No tasks (R)	-21.05	12.27	0.09	-0.43	-13.97	15.86	0.26	-0.25	-49.81	87.85	0.61	-0.14
	Task × route interaction (difference ‘1–3’ – ‘2–4’)	-4.36	26.98	0.87		16.86	24.55	0.49		-109.03	128.90	0.40	
Covariates	Stress	-7.45	9.20	0.42	-0.06/-0.09	5.02	8.67	0.56	0.05	45.65	36.32	0.21	0.09/0.07
	Start time	-14.53/9.98	10.19/6.34	0.15/0.12	-0.13/0.17	18.74^∗^/-3.92	8.13/6.93	0.02/0.57	0.20/-0.06	-16.36	45.82	0.72	-0.04
	Age	-0.06	0.31	0.84	-0.01/-0.02	0.09	0.37	0.81	0.02/0.03	-1.17	1.75	0.50	-0.06/-0.05
	Wayfinding problems	-4.15	19.90	0.84	-0.02/-0.03	-19.55	22.48	0.39	-0.10/-0.12	-272.82^∗^	118.34	0.02	-0.29/-0.24
*R*^2^ (C/R)		0.03/0.06				0.07/0.02				0.12/0.08			


*χ^*2*^ = 9.70 (df = 13, *p* = 0.72), RMSEA < 0.001, CFI = 1.00, TLI = 1.27, SMRM = 0.04. Grouping is based on walking direction: C, clockwise; R, reverse; figures separated by “/” if they differed between the groups. 4 outliers deleted; parameters freed across groups: FFAUS (both halves) regressed on start time. ^∗^*p* < 0.05, ^∗∗^*p* < 0.01, ^∗∗∗^*p* < 0.001.*

Those who participated later in the day (and walked the clockwise route) performed the SART with more FFAUS in the 2nd half of the test, whereas problems with wayfinding were connected to reduced SFAUS after the walk (Table 4). Stress and age were not connected to the refined SART variability measures (Table 4).

The variances explained were low for the FFAUS in the 1st (0.03-0.06%) and the 2nd half (0.02-0.07), merely exceeding the minimum recommended *R*^2^ for practically significant effect of 0.04 (Ferguson, 2009). For the SFAUS, the model explained 0.08-0.12 of the change between the measurements. Altogether four outliers were deleted and two parameters freed to obtain a good fit with the data (Table 4).

##### Sensitivity analyses

In the first sensitivity model for the refined SART variability measures including the 4 outliers deleted from the final model, the model fit was extremely bad in terms of all assessed criteria (for example, CFI = 0.438) and thus we found it meaningless to assess its results. In the second sensitivity model excluding those whose mean RT was > 500 ms, the intercept estimates of SFAUS and FFAUS in the 2nd half were no longer statistically significantly different from 0 for the group who conducted the restoration-enhancement tasks in the reverse order (however, the trend was the same). Therefore, the result that conducting the tasks in the reverse order, but not in clockwise order, improved sustained attention in terms of reduced variability was only partly supported in this analysis.

#### Discussion

Our first main result was that self-reported restoration and valence improved in all conditions but this was not connected to conducting the psychological tasks. Activation remained mostly similar. The second main result was that overall, sustained attention performance, as measured by the number of commission errors, improved after the walk, whereas the speed and stability of responding did not change substantially. Unexpectedly, the participants who completed the restoration-enhancement tasks in the reverse order improved their sustained attention performance (evaluated by reduced commission errors and RT variability) most consistently, whereas those who conducted the tasks in the clockwise order showed no changes in sustained attention. In both ‘no task’ conditions, sustained attention improved only in terms of commission errors. Thus, comparing the two conditions where the restoration-enhancement tasks were conducted in different orders, it appeared that the reverse order was more ideal for attention restoration than the hypothesized, theory-driven order. Based on this consistent finding, we modified the contents of the restoration-enhancement tasks for Study 2.

One limitation of this study was that wayfinding was difficult for some. Those who reported problems with wayfinding (*n* = 15) systematically reported lower levels of restoration and valence after the walk. They also responded more impulsively in their sustained attention task, meaning that they performed the SART with consistently faster RTs, combined with an increased number of commission errors and reduced variability (probably due to the fast speed of responding). The fact that the trail included several crossings (which, nevertheless, were marked with yellow ribbons) and required looking at a map to spot the signposts irritated some participants. Furthermore, taking an incorrect turn and having to return was a nuisance for some, although some found minor wandering around in a new environment inevitable. Most, nevertheless, thought that the trail was well marked and easy to follow.

Another limitation was that the route was different depending on the direction of the walk, which could have affected the results for several reasons. Firstly, when walking the clockwise route, the unpleasant parts of the trail (recent clearings) were toward the end of the walk, whereas in the reverse direction the end was intact coniferous forest. Recently clear-cut forests are generally regarded as unpleasant compared to intact forests or forests that have been cut less invasively (Silvennoinen et al., 2002). In addition to being visually unpleasant, some participants verbally reported feeling upset about the ecological consequences of these clearances. These kinds of reactions to the environment may have shown in their post-walk measurements. Secondly, as the signposts were numbered, the participants who completed the instructions in the reverse order could infer that they were doing them in an “incorrect” order so they were not completely blind to the study conditions. Thirdly, the trail was originally designed to be walked in the clockwise direction and thus, it was marked with arrows and was more intuitive to follow that way. Even though we marked the whole trail with yellow ribbons for this study, we chose not to use arrows pointing in the reverse route to avoid confusion, and it is probable that there was more wayfinding involved when walking the reverse route.

For all the above reasons, the finding that the tasks improved, to some degree, sustained attention performance when they were completed in the reverse order is particularly interesting. We speculate that this may partly have to do with the contents of the final tasks and their congruence with the environment. In the clockwise route, the final task related to general life reflection which may induce all kinds of emotional responses, not solely positive ones (for example, rumination). This type of negative emotional response, especially when combined with the unpleasant scene, may have been the reason for reduced sustained attention restoration; a similar pattern was not found when walking the same route without the tasks. In the reverse route, although more difficult to follow, the end of the trail was more visually pleasant and the final task focused on physical and psychological relaxation. These factors could have induced a more fascinated and calm state and thus, according to attention restoration theory (Kaplan and Kaplan, 1989), lead to better sustained attention when walking this route.

Based on this field experiment, there was no evidence that favored completing the restoration-enhancement tasks in the designed, theory-driven order, although there seemed to be no negative effects of doing these tasks either. It is important to also note that we inspected only short-term effects. For example, reflection may not be restorative in the short-term but it can have a longer-term impact on well-being. To assess any potential longer-term effects on general well-being is, however, outside the scope of this study. Relatedly, we studied single nature visits that may not reveal the full potential of these kind of tasks. For some, it may take more time to “learn” to do the tasks, or more repetition to experience any added benefits on affective or attention restoration (Lymeus et al., 2018).

We would like to note that our participants were more nature-oriented than the general population (evaluated by the number of weekly nature visits). Participation alone required 2.5–3 h, and for most it took much longer because they traveled to the study site from other municipalities in the region. The motive to participate seemed, for many, related to an interest in visiting a new natural environment and/or research on the topic of natural environments. The fact that we found few differences between the participants who completed or did not complete the psychological tasks could also be related to the sample being nature-oriented. Some of the participants in the ‘no task’ conditions said that they had been disappointed because they were instructed not to do the tasks, but that they compensated by focusing on other, pleasant features during the walk (such as spotting new plant species and picking berries and mushroom while walking). It is plausible to assume that some nature-oriented people already know how they like to explore a new (natural) environment and that they are more prone to find elements there that they find interesting and engaging.

### Study 2 – Urban Park

In Study 1 we found that self-reported restoration and valence improved after a forest walk in all groups, regardless of the tasks, whereas for sustained attention, conducting the restoration-enhancement tasks in the reversed order seemed the most beneficial. The aims for Study 2 were to conceptually replicate Study 1, addressing its major limitations, and to investigate the effects of urban nature. The hypotheses were the same as in Study 1 (see The Present Studies).

#### Materials and Methods

Unless otherwise stated, the method was same as in Study 1.

##### Study site

The selected 4-km-long trail was within a popular, well-maintained urban park. The area is commonly referred to as Hatanpää arboretum, as it is a habitat for a vast amount of different tree, bush, and plant species, both native and exotic (City of Tampere, 2017). The park comprises three approximately equal-sized, joined parks, and the selected route went through each of these. The first part of the route went along a lake, and the return route went through the middle of the park. There were few crossings along the route and thus, wayfinding was easier than in Study 1. The surface of the route was mainly flat gravel-paved walkway. All parts of the park are located next to a hospital and a built-up residential/industrial/commercial area and thus, the Corine land cover 25ha (2012) data classifies this area as 121 ‘Industrial or commercial units.’ The measurements were taken at a small office room in a nearby mental health service center, approximately 300 m away from the beginning of the trail. A major improvement to Study 1 was that the environment was the same for everyone as all participants walked the same route in the same direction. This way we could exclude the possibility that differences in wayfinding, aesthetics, or vegetation could influence the results.

##### Participants

A total of 122 working-age people participated in the study in 31 sessions. Initially many more signed up but due to bad weather we had to cancel 13 sessions throughout the summer. Participants were recruited via the project’s Facebook page, by sending invitations to local e-mail lists, by placing posters in notice boards around the city center, and by an online event calendar maintained by the leading regional newspaper. To avoid having a more-than-average nature-oriented sample, we named the study “Walking study” (cf. Study 1 was named “Forest walk study”). Contrary to Study 1, we placed a restriction on age so that all participants would be aged between 18 and 64 years, for clearer generalization and prevention of potential problems with the smart phones. In the adverts, in addition to giving relevant information about the study, we stated that we were looking for volunteer participants who were aged 18–64 years; able to walk 4 km at a slow pace; able to use computers and smart phones; did not use medication that affected their concentration, heart, or psyche; and did not participate in Study 1. In the final sample, one participant was excluded because they conducted only half of the assigned tasks along the trail. The self-reports were missing from two participants and the attention task from one.

Within the participants, visits in the area in the past 6 months varied between 0 and 320, with a mean of 8 visits (median 1). Nature-relatedness, measuring subjective connection with nature, was on average on a moderate level (3.68 on a 1-5 scale, with higher values indicating greater nature-relatedness; Nisbet and Zelenski, 2013).

##### Procedure

In contrast with Study 1, the stressor task was more neutral to avoid a priming effect for nature enjoyment/orientation. The participants were asked to introduce themselves and talk about a hobby they enjoyed. Two project workers guided all experiments.

We gave the participants smartphones (Lenovo A Plus) where they used the mobile application ActionTrack (license provided by the City of Tampere) which gave an audio signal whenever they were close to a “signpost.” The application controlled the order of the tasks so that they could not be completed in a different order than planned, and it allowed us to manipulate the contents of the tasks and to maintain blinding to the study conditions. Using this application required no physical manipulation of the environment, as participants could see the route, the direction of the next task, and their location the whole time they were outdoors. As a back-up, all participants received a paper map with detailed instructions. We instructed them to mainly navigate with the mobile application but if there were problems with it or if they found it disturbing, they could use the paper map and instructions.

The experiment took approximately 2 h per participant, of which the walk duration was 1 h (range between 44 and 97 min). An addition to Study 1 was also that the participants’ pulse was measured the whole time with GPS sports watch (Polar V800) and a heart rate sensor at the chest (Polar H7 belt), and they gave saliva samples before and after the walk. Thus, they were instructed to refrain from heavy exercise and alcohol use 24 h prior to the study, and from using caffeine, food, and nicotine 2 h before the study. In the midpoint of the route, all participants were asked three questions via the mobile phone. These additional measures will be reported elsewhere due to space constraints.

The study was carried out in accordance with the recommendations for “Responsible conduct of research and procedures for handling allegations of misconduct in Finland 2012” by the Finnish advisory board on research integrity (TENK). The protocol was approved by the Regional Ethics Committee of the Tampere University Hospital catchment area. All subjects gave written informed consent in accordance with the Declaration of Helsinki.

##### The psychological instructions

We took into account that in Study 1, the theory-driven restoration-enhancement tasks did not seem to bring added value to any of the affective or attention outcomes when they were conducted in the order they were designed. Instead, these tasks in the reverse order were related to better sustained attention. We noted that in the reverse order, the relaxation tasks became the last and may have affected the respondents positively at the end of the experimental walk. Moreover, in the hypothesized order, the task of reflecting on one’s life was the last and could prime the respondents positively but also negatively, producing rumination and decrease in restoration. Thus, we updated these restoration-enhancement tasks so that they still evolved according to the restoration theories and made sense narratively but so that both beginning and end focused on affective and physiological relaxation. Tasks 1-5 remained exactly as in Study 1, but we modified Tasks 6 and 7. For Task 6, we combined the parts of Tasks 2 and 3 that related to being away and mood enhancement, and the final Task (7) was a short version of Task 1. Overall, then, the first three tasks focused on relaxation and mood enhancement, followed by identifying a favorite place (Task 4), mood relief and mindset recognition (Task 5), forgetting worries and mood enhancement (Task 6), and relaxation in the end (Task 7).

For the control task condition, we chose tasks similar to those used in Duvall’s intervention study (Duvall, 2011, 2013). These alternative tasks focused on different senses (4 tasks) and taking on a new role through which one observes the environment (a magician, a photographer, and a small child; 3 tasks). We matched these tasks to the environment so that, for example, a task instructing one to focus on the sense of smell was located close to the well-maintained rose garden. Like the restoration-enhancement tasks, these ‘awareness-enhancement’ tasks were based on the idea of strengthening engagement and interaction with the environment (Duvall, 2011). The critical difference was that the restoration-enhancement tasks directly aimed to induce a more restored state, both physiologically (for example, “let your shoulders relax”) and psychologically (“feel your mood improve”), whereas the awareness-enhancement tasks focused on engagement and sensory experiences without specifically addressing restoration.

##### Study conditions

As shown in Figure 1, the participants were randomly assigned to three different conditions: a walk without tasks (1/3 of the participants), a walk with the updated theory-driven restoration-enhancement tasks (1/3), and a walk with the awareness-enhancement tasks (1/3).

##### Pre- and post-walk measures and covariates

The self-reported and attention measures were the same as in Study 1. For the ROS, the reliabilities, measured by Cronbach’s α’s, were 0.87 before and 0.89 after the walk. The unadjusted means for each outcome before and after the walk are provided in Appendix F.

*Covariates* were the same as in Study 1 with one addition and some modifications. Based on the changes in the procedure and experiences from Study 1, instead of relying on verbal reports, we asked about the ease of *wayfinding* in the electronic questionnaire after the walk (on a 1-4 scale) and about *navigation method* (1 = ‘mainly with the provided smart phone,’ 2 = ‘with both smartphone and the paper map,’ 3 = ‘mainly with the paper map’). *Stress* in the past 4 weeks (Cohen et al., 1983) had, again, a good reliability (α = 0.83). We also asked in the electronic questionnaire if the participants were *afraid* at any point during the walk and if they encountered anything *unusual* that may have influenced their experience (Gatersleben and Andrews, 2013), followed by an open-ended question, but they were rare or not related to the outcomes (Appendix E in Supplementary Material).

##### Data analysis

The data analyses were the same as in Study 1 (see Data Analysis) except that the multigroup models were fitted to three groups according to the study conditions.

#### Results

##### Self-reported restoration and mood

**FIGURE 5:**
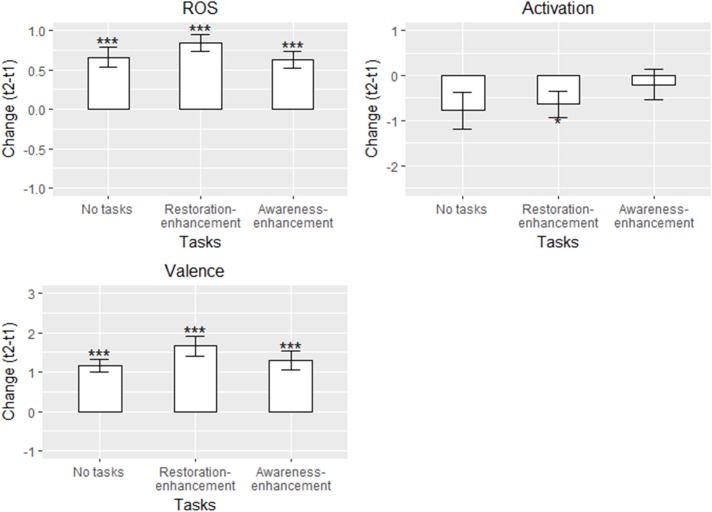
Adjusted means in different conditions for the self-reported measures in Study 2 (*n* = 118). Solid line: statistically significant between-group difference. ^∗^*p* < 0.05, ^∗∗^*p* < 0.01, ^∗∗∗^*p* < 0.001.

As in Study 1, participants in all conditions reported greater restoration and increased valence after the walk, and there were no between-group differences (Figure 5 and Table 5). These findings support our hypothesis 1a but not 2a-c. The estimated changes in self-reported restoration were 0.63-0.84 units, and in valence 1.17-1.66 units. Activation reduced for participants in the ‘no task’ and the updated ‘restoration-enhancement tasks’ conditions (-0.78 to -0.64 units), although this change was statistically significant only in the ‘restoration-enhancement task’ condition (thus, the data showed partial support for hypothesis 1a; Table 5). In the ‘awareness-enhancement tasks’ condition, no changes in activation were apparent.

**Table 5:** The results for multigroup regression models for the self-reported measures in Study 2 (*n* = 118).

		Self-reported restoration				Valence				Activation			
				
		*b*	*SE*	*p*	β	*b*	*SE*	*p*	β	*b*	*SE*	*p*	β
Mean difference, estimated	(1) No tasks	0.66^∗∗∗^	0.13	0.00	0.92	1.17^∗∗∗^	0.16	0.00	1.13	-0.78	0.41	0.06	-0.32
	(2) Restoration-enhancement tasks (U)	0.84^∗∗∗^	0.11	0.00	1.35	1.66^∗∗∗^	0.25	0.00	1.06	-0.64^∗^	0.29	0.03	-0.37
	(3) Awareness-enhancement tasks	0.63^∗∗∗^	0.11	0.00	0.94	1.29^∗∗∗^	0.24	0.00	0.97	-0.20	0.33	0.55	-0.10
Covariates	Stress	0.16	0.11	0.14	0.12/0.12/0.15	0.16	0.22	0.46	0.09/0.05/0.08	0.03	0.37	0.93	0.01/0.01/0.01
	Start time	-0.16	0.17	0.33	-0.10/-0.11/-0.09	0.02	0.26	0.93	0.01/0.01/0.01	-0.65	0.39	0.09	-0.11/-0.16/-0.13
	Age	0.00	0.01	0.80	0.02/0.03/0.02	-0.02	0.01	0.10	-0.20/-0.14/-0.15	-0.01	0.01	0.56	-0.04/-0.06/-0.05
	Navigation method (smart phone – map)	0.12/-0.27/-0.24	0.14/0.15/0.12	0.36/0.06/0.05	0.13/-0.31/-0.27	-0.17	0.15	0.26	-0.12/-0.08/-0.10	-0.24	0.24	0.32	-0.07/-0.10/-0.10
	Ease of wayfinding	0.02	0.11	0.89	0.01/0.02/0.01	0.03	0.22	0.88	0.02/0.02/0.01	0.42	0.36	0.24	0.09/0.17/0.11
*R*^2^ (conditions 1/2/3)		0.04/0.12/0.11				0.08/0.04/0.05				0.03/0.06/0.04			


*χ^2^ = 26.36 (df = 34, *p* = 0.82), RMSEA < 0.001, CFI = 1.00, TLI = 1.73, SMRM = 0.08. Grouping is based on the study condition; figures separated by “/” if they differed between the groups. U, updated from Study 1. Parameters freed across groups: ROS regressed on navigation method. ^∗^*p* < 0.05, ^∗∗^*p* < 0.01, ^∗∗∗^*p* < 0.001.*

Stress, start time, age, and ease of wayfinding were not connected to the changes in the self-reported outcomes (Table 5). Using the paper map instead of smart phone was connected to a smaller change in self-reported restoration in the conditions where participants conducted tasks (Table 5).

Altogether, the *R*^2^s were lower than in Study 1, although in self-reported restoration and valence they mainly exceeded 0.04, the recommended minimum cut-off for practical significance (Ferguson, 2009). In activation, *R*^2^s varied between 0.03 and 0.06. The model fit was good with one parameter freed (Table 5).

##### SART – traditional measures

**FIGURE 6:**
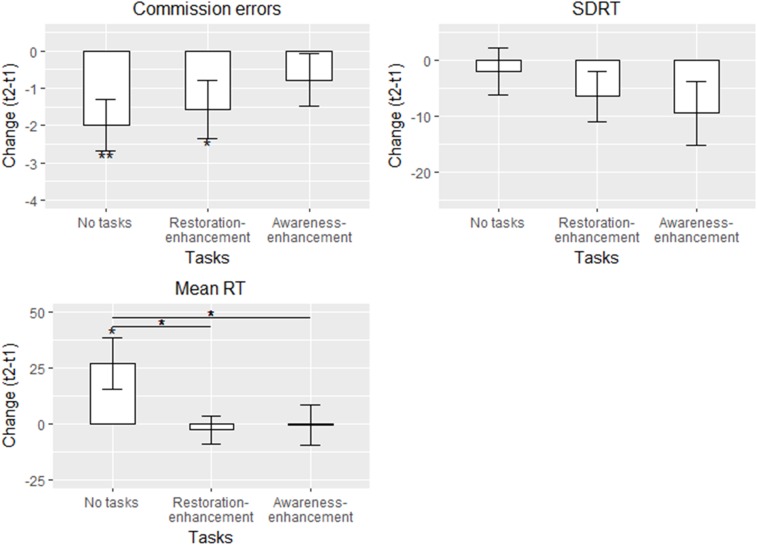
Adjusted means in different conditions for the traditional SART measures in Study 2 (*n* = 116). Solid line: statistically significant between-group difference. ^∗^*p* < 0.05, ^∗∗^*p* < 0.01, ^∗∗∗^*p* < 0.001.

Participants in the ‘no tasks’ and ‘restoration-enhancement tasks’ conditions made 1.57 - 1.99 less commission errors after the walk compared with before (Figure 6 and Table 6), whereas for those in the awareness-enhancement tasks condition, the trend was in the same direction but not significant (partially supporting hypothesis 1b). Mean RT slowed on average by 27 ms for the ‘no task’ group, whereas no changes were apparent in the other conditions, contrasting hypothesis 1b but supporting hypothesis 2b. For SDRT, against all our hypotheses, none of the groups showed change between the measurements.

**Table 6:** The results for multigroup regression models for the traditional SART measures in Study 2 (*n* = 116).

		Commission errors				Mean RT (ms)				SDRT (ms)			
				
		*b*	*SE*	*p*	β	*B*	*SE*	*p*	β	*B*	*SE*	*p*	β
Mean difference, estimated	(1) No tasks	-1.99^∗∗^	0.70	0.00	-0.47	27.04^∗^	11.53	0.02	0.46	-1.91	4.22	0.65	-0.08
	(2) Restoration-enhancement tasks (U)	-1.57^∗^	0.79	0.05	-0.32	-2.58	6.28	0.68	-0.06	-6.43	4.45	0.15	-0.27
	(3) Awareness-enhancement tasks	-0.78	0.71	0.27	-0.18	-0.26	8.95	0.98	-0.01	-9.41	5.71	0.10	-0.30
Covariates	Stress	-1.75^∗^/2.51/49	0.77/1.38/0.77	0.02/0.07/0.53	-0.23/0.25/0.07	-16.03^∗^	7.83	0.04	-0.15/-0.17/-0.20	-8.73^∗^	4.48	0.05	-0.20/-0.18/-0.17
	Start time	2.74^∗∗^	0.85	0.00	0.27/0.22/0.24	-61.86^∗∗∗^/-33.67^∗^/-14.47	12.81/14.33/13.25	0.00/0.02/0.28	-0.45/-0.30/-0.11	-24.01^∗∗^/-1.61/0.94	7.62/5.97/12.31	0.00/0.79/0.94	-0.41/-0.03/0.01
	Age	-0.01	0.04	0.78	-0.03/-0.02/-0.03	-0.43/0.79/-0.97	0.69/0.53/0.61	0.53/0.14/0.12	-0.08/0.19/-0.20	-0.38/0.36/-0.28	0.36/0.31/0.36	0.30/0.25/0.44	-0.17/0.17/-0.09
	Navigation method (smart phone - map)	1.30^∗∗^	0.48	0.01	0.23/0.17/0.23	4.83/-25.36^∗∗^/5.96	7.83/8.99/8.68	0.54/0.01/0.49	0.06/-0.36/0.09	-3.16	2.95	0.28	-0.09/-0.08/-0.08
	Ease of wayfinding	-0.67	0.72	0.35	-0.08/-0.09/-0.08	9.93	6.95	0.15	0.08/0.15/0.10	4.50	3.70	0.23	0.09/0.13/0.07
*R*^2^ (conditions 1/2/3)		0.23/0.09/0.13				0.20/0.16/0.06				0.24/0.07/0.04			


*χ^*2*^ = 20.08 (df = 22, *p* = 0.58), RMSEA < 0.001, CFI = 1.00, TLI = 1.03, SMRM = 0.06. Grouping is based on the study condition; figures separated by “/” if they differed between the groups. U, updated from Study 1. 1 outlier deleted; parameters freed across groups: RT and SDRT regressed on age and start time, RT regressed on navigation method, commission errors regressed on stress, covariance between RT and SDRT. ^∗^*p* < 0.05, ^∗∗^*p* < 0.01, ^∗∗∗^*p* < 0.001.*

Those who had experienced more stress in the past 4 weeks made less commission errors (in the ‘no tasks’ condition only) and responded faster after the walk compared to before (all conditions; Table 6). Start time was associated with most of the measures of sustained attention: those who participated in the afternoon made more commission errors in all groups, responded faster (in two conditions), and there was less variability in their response times (in the ‘no tasks’ condition) after the walk (Table 6). Using the map instead of the smart phone for navigation was connected to an increased number of commission errors (all groups) and to a speeding of mean RT (in the ‘restoration-enhancement tasks’ condition). Age was not connected to the changes in the outcomes.

The variances explained were consistently highest in the ‘no task’ condition (0.20-0.24) and lower and more variable in the other conditions, yet exceeding the 0.04 threshold for practical significance. Initially, the model fit was very bad but improved after freeing seven parameter estimates across the groups (Table 6).

##### SART – refined variability measures

**FIGURE 7:**
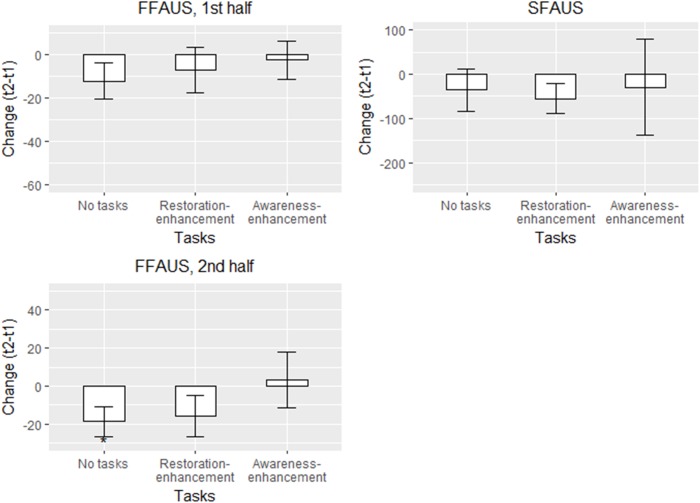
Adjusted means in different conditions for the refined SART variability measures in Study 2 (*n* = 113). Solid line: statistically significant between-group difference. ^∗^*p* < 0.05, ^∗∗^*p* < 0.01, ^∗∗∗^*p* < 0.001.

In the first half of the SART, against hypotheses 1b and 2a - c, no changes in FFAUS were apparent after the walk in any of the conditions (Figure 7 and Table 7). In the second half, the participants in the ‘no tasks’ condition performed the task with less FFAUS; the trend was similar for participants who conducted the updated restoration-enhancement tasks but there was more variability within the group (showing partial support for hypothesis 1b but contrasting hypotheses 2a-c; Table 7). In terms of SFAUS, no changes occurred within or between the groups (against all hypotheses).

**Table 7:** The results for multigroup regression models for the refined SART variability measures in Study 2 (*n* = 113).

		FFAUS, 1st half				FFAUS, 2nd half				SFAUS				
				
		*b*	*SE*	*p*	β	*b*	*SE*	*p*	β	*b*	*SE*	*p*	β
Mean difference, estimated	(1) No tasks	-12.26	8.26	0.14	-0.29	-18.71^∗^	7.81	0.02	-0.39	-35.73	48.18	0.46	-0.16
	(2) Restoration-enhancement tasks (U)	-7.15	10.32	0.49	-0.11	-15.81	10.78	0.14	-0.23	-55.58	33.44	0.10	-0.28
	(3) Awareness-enhancement tasks	-2.55	8.72	0.77	-0.05	3.08	14.64	0.83	0.04	-29.77	107.98	0.78	-0.06
Covariates	Stress	-15.88	10.37	0.13	-0.21/-0.12/-0.20	-25.77^∗^	12.66	0.04	-0.31/-0.18/-0.20	-52.55	48.73	0.28	-0.14/-0.13/-0.06
	Start time	-27.29^∗^	11.59	0.02	-0.26/-0.17/-0.20	6.11	14.57	0.68	0.05/0.04/0.03	-38.73/90.54/12.57	59.36/66.77/111.53	0.51/0.18/0.91	-0.07/0.19/0.01
	Age	-0.46	0.47	0.33	-0.12/-0.08/-0.09	-0.43	0.51	0.40	-0.10/-0.07/-0.06	-1.48	2.36	0.53	-0.07/-.08/-0.03
	Navigation method	0.81	6.85	0.91	0.01/0.01/0.01	-15.25/-12.47/23.09	8.23/15.45/15.83	0.06/0.42/0.15	-0.24/-0.12/0.22	12.72	27.98	0.65	0.04/0.04/0.02
	Ease of wayfinding	5.91	9.12	0.52	0.07/0.07/0.06	3.91	9.18	0.67	0.04/0.04/0.02	29.53	36.21	0.42	0.07/0.11/0.03
*R*^2^ (conditions 1/2/3)		0.09/0.05/0.06				0.16/0.05/0.08				0.03/0.08/0.004				


*χ^2^ = 26.36 (df = 34, *p* = 0.82), RMSEA < 0.001, CFI = 1.00, TLI = 1.73, SMRM = .08. Grouping is based on the study condition; figures separated by “/” if they differed between the groups. U: updated from Study 1. 3 outliers deleted; parameters freed across groups: FFAUS (2nd half) regressed on navigation method and SFAUS regressed on start time. ^∗^*p* < 0.05, ^∗∗^*p* < 0.01, ^∗∗∗^*p* < 0.001.*

Age, navigation method, and ease of wayfinding were not connected to the changes in the refined SART variability measures. Participants who were more stressed performed the second half of the SART with less FFAUS after the walk (Table 7). Similarly, later start time predicted less FFAUS in the first half of the test.

Variances explained varied between 0.05 and 0.16 in FFAUS, exceeding the threshold for practical significance, but in SFAUS, the *R*^2^s were poor (0.004-0.08). As in Study 1, the model for these outcomes had several large outliers, 3 of which were deleted (Table 7). In addition, 2 parameters were freed across groups.

##### Sensitivity analyses

In the sensitivity model for the traditional SART measures including the outlier deleted from the final model, the greatest difference to the final model was that more stress in the past 4 weeks was connected to lower SDRT. No substantial differences in other estimates, their significance levels or in the conclusions drawn from them were apparent.

Similarly, in the sensitivity model for the refined variability measures including the 3 outliers deleted from the final model, the only substantial difference to the reported model was that more stress predicted less FFAUS also in the 1st half of the test. In the second sensitivity model excluding the participants whose mean RT was > 500 ms, the only substantial difference to the final model was that the participants who conducted the restoration-enhancement tasks showed lower FFAUS in the 2nd half. This result strengthens our conclusion that sustained attention improved in this condition.

#### Discussion

Consistent with Study 1, self-reported restoration and valence increased after the walk in all conditions. In addition, participants were generally more relaxed after the walk compared to before. No differences between the three groups were found on these self-reported measures, however. In terms of sustained attention performance, the participants who conducted the updated restoration-enhancement tasks made less commission errors after the walk but there was no change in their mean RT or SDRT. This indicates an improvement in response accuracy, attention control, and response inhibition following restoration-enhancement but no effect on their speed or variability in responding. For those who conducted the awareness-enhancement tasks, no changes in sustained attention performance were detected. The participants who did not conduct the tasks made less commission errors but their mean RT slowed significantly more than in the other conditions. They also showed less moment-to-moment variability in responding (FFAUS) in the 2nd half of the SART after the walk. Thus, like Study 1, in terms of sustained attention, conducting the restoration-enhancement tasks resulted in greatest improvements in sustained attention performance, followed by walking without tasks.

Although using the smart phones instead of reading the tasks from signposts improved the procedure from Study 1, some found the smart phones disturbing. Being irritated about having to use the smart phone and resorting to using the map could explain why using the paper map was consistently associated with lower self-reported restoration and increased number of SART commission errors (and, in some groups, faster response time). As we instructed the participants to primarily navigate with the smart phones, unless they found it disturbing, it is plausible that using the paper map was a result of being irritated during the walk. Relatedly, the participants who conducted tasks had to use the smart phone inevitably more throughout the walk: they viewed the tasks’ locations, listened to the signals, and read the tasks from the screen. Having to use the smart phone more could have hindered the quality of interaction with the environment, however, our results indicate no such case. The responses between the ‘no tasks’ and ‘restoration-enhancement tasks’ conditions were, in fact, very similar with few exceptions.

Both stress and start time were connected to attention restoration but in opposite ways. Later start time was consistently related to more impulsive responding during the SART, that is, faster responding and making more commission errors. This could be explained by the circadian rhythm and attention fatigue during the day (Riley et al., 2017), as usually those who participated later came directly after work. Being more stressed in the past 4 weeks was also connected to responding faster but making less commission errors and having less moment-to-moment attentional slips toward the end of the sustained attention test. Thus, the results indicate that participants who were more stressed experienced more sustained attention restoration during the nature walk whereas sustained attention was not restored after participating later during the day (and possibly after work).

It is important to note that even though we found no evidence that the awareness-enhancement tasks improved attention restoration, they were used very differently than in Duvall’s original studies (Duvall, 2011, 2013). In these studies, the participants could choose which tasks to use and when; they could change the tasks frequently between or within their walks, or keep on doing the same task during multiple walks. Duvall’s intervention (Duvall, 2011, 2013) covered several nature walks during 2 weeks, and it is possible that some restorative effects reported in these interventions may develop over longer time periods because participants may need more time to learn and become used to the tasks (Lymeus et al., 2018).

## Discussion

### Overall Discussion (Studies 1 and 2)

Our experimental field studies support the established findings that various types of nature visits enhance positive mood but the effects on attention restoration are more nuanced (McMahan and Estes, 2015; Ohly et al., 2016). Although our studies varied in exposure time and environmental quality, the self-reported mood-related outcomes, valence and restoration, showed a similar, positive change. This is in line with meta-analyses summarizing experimental studies on nature exposure (Barton and Pretty, 2010; McMahan and Estes, 2015). Sustained attention improved overall in terms of reduced commission errors; this can indicate less mindlessness and fewer attentional slip-ups in ‘real life’ (Robertson et al., 1997). The fact that there were fewer differences between self-reported outcomes compared to sustained attention corroborates findings from Lin et al. (2014). In both our studies, the greatest improvements in sustained attention were experienced when the participants conducted the restoration-enhancement tasks ending with instructed relaxation. Less clear, however, is the longevity of these effects, and potential benefits over repeated walks. Repeated exposure to, and engagement with, a natural environment could provide added restoration via place attachment and favorite place establishment (Korpela et al., 2010). We have seen encouraging results showing the attention benefits of repeatedly engaging with the environment via different types of engagement strategies (Duvall, 2011; Lymeus et al., 2018). Whether the psychological tasks examined in our studies could provide similar benefits over a longer course is a matter for future research. Furthermore, as our studies integrated components of different restoration mechanisms (attention restoration, stress reduction, and place attachment), future research investigating the relative contributions of these components in providing restorative outcomes would be worthwhile.

The finding that both mood and sustained attention improved after a nature walk not only supports Stress reduction theory and Attention restoration theory but also the idea that the processes they describe are co-occurring (Kaplan, 1995; Markevych et al., 2017). This was further supported by the strong role of stress prior to, and during, the experiment in explaining both changes in affective and attention restoration. The role of environmental engagement in enhancing restorative benefits of nature exposure, on the other hand, is less clear. We found evidence that restoration-enhancement tasks, aimed to guide interaction with the environment, can aid sustained attention but no indication that it could enhance affective restoration. Furthermore, there was no evidence (in Study 1) that to promote sustained attention, the tasks should follow the theory-based sequence with life reflection at the final stage, or that tasks focusing on engagement without addressing restoration would benefit sustained attention (Study 2; cf. Duvall, 2011). The fact that the contents and the order of the tasks and their congruence with the environment mattered in terms of sustained attention highlights the sensitive and complex nature of person-environment interaction (Kaplan and Kaplan, 1989). Our understanding of these complexities might benefit from qualitative future investigation. Furthermore, although our results suggest that engagement with the environment can be a relevant facilitator of attention restoration, it is, naturally, possible that other type of tasks or forms of engagement could promote both attention and affective restoration more effectively, or, consistently.

Our studies were conducted in the field with a focus on creating a realistic nature visit. It is expected that people respond to these types of psychological tasks differently, and in both our studies, participants could complete them in a way they preferred. Concurrently, this means that we had little control over how ‘well’ the tasks were conducted, how much time was spent on the tasks, or on the quality of the environmental interaction that the tasks aimed to enhance. To better understand restoration process and the relative contributions of each component in the restoration process – physiological, affective, attentional – it would have been useful to have a measure to assess interaction with the environment during the walk, and not just the restorative outcomes following it. However, examining person-environment interaction without disturbing this interaction could be challenging, and it remains a topic for future studies to explore. Similarly, the fact that the participants could walk at their own pace improved the external validity of the experiment but, at the same time, we could not control for events during the walk (Abrahamse et al., 2016). Had the participants walked in groups, the presence of others, the group size, or inability to walk at one’s typical pace may have also affected the experiment in a more positive or negative way (e.g., Staats and Hartig, 2004).

Because the two studied paths differed in environmental type, length, and signing, we conducted no analyses comparing the effects between the studies. Overall, however, the effects of these two similar experiments were to the same direction in all our measures. This gave us more confidence to draw conclusions, especially when conclusions from the individual studies had to be made with caution due to lower-than-planned sample sizes and, consequently, less power in the statistical analyses. The fact that the findings were similar in the two studies accords with a number of studies and meta-analyses that have found no difference between the restorative effects of wild and maintained natural environments, or otherwise different types of natural environments (Barton and Pretty, 2010; McMahan and Estes, 2015; Rogerson et al., 2016).

Finally, it is important to note that our results may not apply to the general population. Although the samples had the benefit of being more diverse than the commonly used student samples, the participants were mostly female and likely more nature-oriented than the general population. To obtain more diverse samples, similar future studies could try different recruitment methods (such as targeting employees near the study sites) and providing more incentives (such as raffles or more extensive feedback) for participation. Another issue with the samples were drop-outs due to last-minute cancelations and bad weather. The cancelation rates were smaller in Study 2 that, compared to Study 1, was shorter, more easily accessible by public transport, and used an online-calendar for signing up in the study; all these features probably contributed to lower sample attrition and could be recommended for future studies.

## Conclusion

Our studies focused on the concept of active engagement with the environment, previously receiving scant empirical attention, advancing our theoretical and practical understanding of the restorative environments field. We examined this by designing, and testing, the effects of restoration-enhancement tasks along nature trails. The present studies indicate that these tasks can have a beneficial influence on sustained attention, whereas self-reported restoration and valence appear to improve after a nature walk regardless of conducting tasks. The studies also provide tentative evidence that the effects on sustained attention are sensitive to the tasks’ contents: conducting tasks can either hinder or facilitate performance in a sustained attention task compared with regular nature walks without tasks. These findings are in line with both Stress reduction theory and Attention restoration theory, and support the idea that these two theories about attention and affective restoration describe complementary processes (Kaplan, 1995; Markevych et al., 2017).

Most Finnish people regularly spend time in nature, and the most common recreational activity in nature is walking (Sievänen and Neuvonen, 2011). It is also common to visit natural settings for stress reduction purposes and to experience restoration from such visits (Pasanen et al., 2018). Our studies indicate that some aspects of restoration during nature walks could be enhanced by encouraging active engagement with the environment. We already have tentative evidence that self-reported restoration evaluations are similar across visits to nature trails with the same tasks in other European countries (Korpela et al., 2017). Transferring these tasks to other countries and routes is low-cost and requires little-to-no physical environmental modification, and promoting their use has, thus, potentially wider benefits. Moreover, conducting restoration-enhancement tasks or other engagement strategies during a nature walk is free for the public, and it may facilitate interaction with the surrounding environment, especially in cases where natural settings are less optimal, uninteresting or cannot be easily redesigned (cf. Duvall, 2011). Ideally, the tasks could support nature visitors’ everyday attention restoration, enhance motivation to visit restorative (natural) settings, and educate or sensitize people who are not familiar with interacting with nature. Restoration-enhancement tasks are, in conclusion, a promising avenue for enhancing the benefits of nature experiences.

## Author Contributions

This study was originated by KK, who designed and planned the experiments with TP. TP collected the data with a research assistant, conducted the statistical analyses, and wrote the majority of the paper. KJ calculated the variables for FFAUS and SFAUS. KK, KL, and KJ critically revised the manuscript several times. All authors contributed to data interpretation and gave final approval to the version to be published.

## Conflict of Interest Statement

The authors declare that the research was conducted in the absence of any commercial or financial relationships that could be construed as a potential conflict of interest. The reviewer JA and handling Editor declared their shared affiliation.
