# Supplementary material for: Can Nature Walks With Psychological Tasks Improve Mood, Self-Reported Restoration, and Sustained Attention? Results From Two Experimental Field Studies
Source: Front Psychol. 2018 Oct 30;9:2057. doi: 10.3389/fpsyg.2018.02057 (PMC6218585; doi:10.3389/fpsyg.2018.02057)
Supplement: Supplementary file 1 [file Table_1.docx]

Supplementary Material

Can nature walks with psychological tasks improve mood, self-reported restoration, and sustained attention? Results from two experimental field studies.

Tytti Pasanen*, Katherine Johnson, Kate Lee, Kalevi Korpela

*** Correspondence:** Corresponding Author: tytti.pasanen@uta.fi

# Supplementary Tables

**Appendix A** Correlations between the change in the outcomes and the potential continuous covariates in Study 1. Upper diagonal: Spearman correlations; lower diagonal: *p*-values

|  | Restoration | Valence | Activation | SART commission errors | SART RT | SART SDRT | SART SFAUS | SART FFAUS, 1st half | SART FFAUS, 2nd half | Temperature | Walk duration (min) | Stress | Age | Sleep (h) |
| --- | --- | --- | --- | --- | --- | --- | --- | --- | --- | --- | --- | --- | --- | --- |
| Restoration |  | .55 | .02 | -.10 | .03 | -.08 | .02 | .14 | .03 | .11 | -.07 | .25 | -.27 | -.06 |
| Valence | .00 |  | -.17 | -.03 | -.03 | -.13 | .05 | .13 | .10 | .03 | -.13 | .15 | -.13 | -.09 |
| Activation | .86 | .06 |  | .01 | -.04 | -.08 | .18 | -.08 | -.04 | -.08 | .01 | -.06 | .00 | -.02 |
| SART commission errors | .25 | .71 | .93 |  | -.48 | .01 | .04 | -.15 | -.07 | .06 | .04 | -.13 | .01 | -.03 |
| SART RT | .71 | .77 | .67 | .00 |  | .55 | -.44 | -.08 | -.20 | -.08 | -.09 | .12 | -.07 | .11 |
| SART SDRT | .38 | .16 | .39 | .90 | .00 |  | -.67 | -.51 | -.58 | .15 | .02 | -.07 | -.10 | .17 |
| SART SFAUS | .82 | .56 | .04 | .64 | .00 | .00 |  | .27 | .30 | -.13 | .00 | -.02 | .13 | -.15 |
| SART FFAUS, 1st half | .12 | .15 | .39 | .11 | .41 | .00 | .00 |  | .46 | .00 | .01 | .11 | .07 | -.08 |
| SART FFAUS, 2nd half | .73 | .27 | .63 | .43 | .03 | .00 | .00 | .00 |  | -.07 | .12 | .15 | .20 | -.07 |
| Temperature | .21 | .78 | .39 | .50 | .38 | .11 | .15 | .98 | .48 |  | .12 | -.06 | .00 | -.05 |
| Walk duration (min) | .41 | .13 | .90 | .66 | .31 | .85 | .98 | .91 | .20 | .16 |  | .06 | .20 | -.10 |
| Stress | .00 | .08 | .47 | .16 | .18 | .46 | .79 | .24 | .11 | .49 | .49 |  | -.18 | -.20 |
| Age | .00 | .15 | .96 | .93 | .43 | .27 | .14 | .42 | .03 | .97 | .03 | .04 |  | -.09 |
| Sleep (h) | .49 | .33 | .82 | .76 | .21 | .05 | .09 | .38 | .42 | .57 | .26 | .02 | .29 |  |

**Appendix B** ANOVAs for potential categorical covariates and the outcomes in Study 1

|  | | Start time | | Weather | | Gender | | Problems with wayfinding | |
| --- | --- | --- | --- | --- | --- | --- | --- | --- | --- |
| Outcome (change t2-t1) | | F-test | p | F-test | p | F-test | p | F-test | p |
| Restoration | | F(2,128)=4.81 | .01 | F(3,128)=.47 | .70 | F(1,127)=0 | 1.00 | F(1,128)=17.04 | <.01 |
| Valence | | F(2,127)=3.11 | .05 | F(3,127)=.21 | .89 | F(1,126)=.19 | .66 | F(1,127)=8.86 | <.01 |
| Activation | | F(2,127)=.27 | .77 | F(3,127)=2.62 | .05 | F(1,126)=.83 | .36 | F(1,127)=0 | .95 |
| SART | Commission errors | F(2,124)=.5 | .61 | F(3,124)=.2 | .90 | F(1,123)=.05 | .82 | F(1,124)=6.99 | .01 |
|  | RT | F(2,122)=1.35 | .26 | F(3,122)=.74 | .53 | F(1,121)=.6 | .44 | F(1,122)=8.65 | <.01 |
|  | SDRT | F(2,122)=.89 | .41 | F(3,122)=.62 | .60 | F(1,121)=.01 | .92 | F(1,122)=3.95 | .05 |
|  | FFAUS, 1st half | F(2,121)=.51 | .60 | F(3,121)=.02 | 1.00 | F(1,120)=.01 | .92 | F(1,121)=2.87 | .09 |
|  | FFAUS, 2nd half | F(2,121)=.08 | .92 | F(3,121)=.81 | .49 | F(1,120)=.09 | .77 | F(1,121)=.82 | .37 |
|  | SFAUS | F(2,121)=.38 | .69 | F(3,121)=.87 | .46 | F(1,120)=.75 | .39 | F(1,121)=11.73 | <.01 |

**Appendix C** Unadjusted means and standard deviations (*SD*) before and after the walk in Study 1, grouped by study condition.

|  |  |  |  |  |  |  |  |  | Sustained attention to response task | | | | | | | | | | | |
| --- | --- | --- | --- | --- | --- | --- | --- | --- | --- | --- | --- | --- | --- | --- | --- | --- | --- | --- | --- | --- |
| Condition | |  | Restoration | | Valence | | Activation | | Commission errors | | RT | | SDRT | | FFAUS, 1st half | | FFAUS, 2nd half | | SFAUS | |
| Route | Tasks |  | Before | After | Before | After | Before | After | Before | After | Before | After | Before | After | Before | After | Before | After | Before | After |
| Clockwise | No | Mean | 4.88 | 5.2 | 5.96 | 7.71 | 6 | 4.71 | 9.3 | 7.52 | 427.87 | 400.39 | 109.75 | 94.46 | 104.7 | 82.99 | 84.97 | 88.33 | 443.55 | 376.19 |
|  |  | *N* | 24 | 24 | 24 | 24 | 24 | 24 | 23 | 23 | 22 | 23 | 22 | 23 | 21 | 22 | 21 | 22 | 21 | 22 |
|  |  | *SD* | 0.65 | 0.69 | 1.85 | 0.91 | 1.22 | 1.49 | 4.82 | 4.64 | 74.59 | 60.09 | 33.27 | 35.59 | 61.4 | 70.7 | 44.91 | 70.49 | 374.18 | 434.8 |
| Reverse | No | Mean | 4.56 | 5.03 | 6.06 | 7.18 | 5.53 | 5.76 | 7.29 | 5.47 | 409.01 | 430.41 | 92.04 | 81.66 | 98 | 72.92 | 82.78 | 68.91 | 378.68 | 322.3 |
|  |  | *N* | 17 | 17 | 17 | 17 | 17 | 17 | 17 | 17 | 17 | 17 | 17 | 17 | 17 | 17 | 17 | 17 | 17 | 17 |
|  |  | *SD* | 0.85 | 0.77 | 1.82 | 1.33 | 1.5 | 1.71 | 4.83 | 5.14 | 89.58 | 97.83 | 31.9 | 35.44 | 65.17 | 53.17 | 46.82 | 68.31 | 345.22 | 374.43 |
| Clockwise | Yes | Mean | 4.61 | 5.21 | 6.2 | 7.58 | 5.36 | 4.93 | 9.59 | 8.93 | 398.85 | 404.7 | 102.64 | 97.43 | 91.06 | 97.65 | 103.65 | 93.31 | 373.12 | 377.81 |
|  |  | *N* | 45 | 45 | 45 | 45 | 45 | 45 | 44 | 44 | 44 | 44 | 44 | 44 | 40 | 41 | 40 | 41 | 40 | 41 |
|  |  | SD | 0.96 | 0.87 | 1.8 | 1.14 | 1.54 | 1.78 | 4.99 | 5.57 | 76.06 | 100.19 | 42.8 | 38.79 | 68.59 | 100.57 | 81.4 | 69.14 | 428.88 | 318.71 |
| Reverse | Yes | Mean | 4.84 | 5.19 | 6.19 | 7.21 | 5.79 | 5.31 | 9.51 | 8.66 | 418.69 | 401.74 | 105.39 | 92.46 | 94.47 | 87.58 | 109.49 | 85.85 | 518.09 | 359.85 |
|  |  | *N* | 43 | 43 | 43 | 42 | 43 | 42 | 41 | 41 | 40 | 40 | 40 | 40 | 40 | 40 | 40 | 40 | 40 | 40 |
|  |  | *SD* | 0.83 | 0.82 | 1.62 | 1.54 | 1.68 | 1.63 | 5.34 | 4.46 | 77.43 | 72.14 | 39.26 | 34.21 | 65.71 | 67.04 | 71.09 | 49.87 | 471.79 | 293 |
| Total | | Mean | 4.73 | 5.18 | 6.13 | 7.43 | 5.64 | 5.13 | 9.2 | 8.11 | 411.9 | 406.47 | 103.34 | 93.11 | 95.65 | 88.1 | 99.3 | 86.45 | 435.59 | 363.66 |
|  |  | *N* | 129 | 129 | 129 | 128 | 129 | 128 | 125 | 125 | 123 | 124 | 123 | 124 | 118 | 120 | 118 | 120 | 118 | 120 |
|  |  | *SD* | 0.85 | 0.8 | 1.74 | 1.28 | 1.54 | 1.68 | 5.06 | 5.08 | 78.03 | 84.53 | 38.59 | 36.23 | 65.24 | 78.77 | 68.24 | 63.14 | 424.13 | 338.88 |

**Appendix D** Correlations between the change in the outcomes and the potential continuous covariates in Study 2. Upper diagonal: Spearman correlations; lower diagonal: *p*-values

|  | Restoration | Valence | Activation | SART commission errors | SART RT | SART SDRT | SART SFAUS | SART FFAUS, 1st half | SART FFAUS, 2nd half | Temperature | Walk duration (min) | Stress | Age | Sleep (h) |
| --- | --- | --- | --- | --- | --- | --- | --- | --- | --- | --- | --- | --- | --- | --- |
| Restoration |  | .47 | -.01 | -.15 | .15 | .06 | .08 | .13 | -.18 | -.04 | .12 | .17 | -.01 | -.10 |
| Valence | .00 |  | .01 | -.20 | .14 | -.05 | -.01 | .00 | -.20 | -.10 | .09 | .14 | -.18 | -.15 |
| Activation | .96 | .92 |  | -.14 | .13 | .08 | .06 | .04 | -.07 | -.03 | .04 | .06 | -.09 | -.17 |
| SART commission errors | .10 | .03 | .13 |  | -.67 | -.14 | -.03 | -.06 | .04 | .14 | .02 | .00 | .03 | -.07 |
| SART RT | .10 | .12 | .16 | .00 |  | .48 | .35 | .31 | .17 | -.13 | -.02 | -.09 | -.06 | .12 |
| SART SDRT | .51 | .62 | .38 | .13 | .00 |  | .79 | .54 | .58 | .02 | .09 | -.15 | .01 | .11 |
| SART SFAUS | .40 | .87 | .50 | .76 | .00 | .00 |  | .51 | .43 | -.01 | .06 | -.08 | -.01 | .14 |
| SART FFAUS, 1st half | .17 | .96 | .65 | .51 | .00 | .00 | .00 |  | .23 | -.07 | .01 | -.10 | -.06 | .15 |
| SART FFAUS, 2nd half | .05 | .03 | .43 | .64 | .07 | .00 | .00 | .01 |  | .08 | .03 | -.19 | -.08 | .13 |
| Temperature | .69 | .30 | .71 | .13 | .16 | .84 | .91 | .45 | .36 |  | .10 | -.06 | .10 | .07 |
| Walk duration (min) | .21 | .32 | .67 | .81 | .86 | .32 | .54 | .93 | .72 | .26 |  | -.02 | .27 | .02 |
| Stress | .08 | .13 | .49 | .96 | .35 | .11 | .36 | .28 | .04 | .49 | .81 |  | -.25 | .01 |
| Age | .89 | .05 | .31 | .77 | .54 | .90 | .87 | .52 | .37 | .30 | .00 | .01 |  | -.08 |
| Sleep (h) | .27 | .10 | .06 | .44 | .21 | .25 | .12 | .11 | .15 | .47 | .80 | .89 | .40 |  |

**Appendix E** ANOVAs for potential categorical covariates and the outcomes in Study 2

|  |  | Start time | | Weather | | Gender | | Navigation method | | Ease of wayfinding | | Fear | | Unusual event | |
| --- | --- | --- | --- | --- | --- | --- | --- | --- | --- | --- | --- | --- | --- | --- | --- |
| Outcome (change t2-t1) | | F-test | p | F-test | p | F-test | p | F-test | p | F-test | p | F-test | p | F-test | p |
| Restoration | | F(1,116)=.26 | .61 | F(3,116)=.74 | .53 | F(2,116)=1.07 | .35 | F(2,116)=1.88 | .16 | F(2,116)=.02 | .98 | F(1,116)=0 | .97 | F(2,116)=2.44 | .09 |
| Valence | | F(1,119)=.14 | .71 | F(3,119)=1.27 | .29 | F(2,119)=3.3* | .04 | F(2,119)=1.36 | .26 | F(2,119)=.69 | .51 | F(1,119)=.36 | .55 | F(2,119)=.18 | .84 |
| Activation | | F(1,119)=3.79 | .05 | F(3,119)=1.1 | .35 | F(2,119)=1.28 | .28 | F(2,119)=.84 | .43 | F(2,119)=.31 | .74 | F(1,119)=.79 | .38 | F(2,119)=.01 | .99 |
| SART | Commission errors | F(1,119)=6.96 | .01 | F(3,119)=1.18 | .32 | F(2,119)=.56 | .57 | F(2,119)=3.49 | .03 | F(2,119)=.11 | .89 | F(1,119)=.19 | .66 | F(2,119)=.81 | .45 |
|  | RT | F(1,119)=7.81 | .01 | F(3,119)=2.14 | .10 | F(2,119)=.70 | .50 | F(2,119)=.44 | .64 | F(2,119)=.11 | .90 | F(1,119)=.10 | .75 | F(2,119)=1.45 | .24 |
|  | SDRT | F(1,119)=3.89 | .05 | F(3,119)=.7 | .55 | F(2,119)=.17 | .85 | F(2,119)=1.63 | .20 | F(2,119)=.34 | .71 | F(1,119)=2.85 | .09 | F(2,119)=.52 | .60 |
|  | FFAUS, 1st half | F(1,117)=1.78 | <.01 | F(3,117)=7.92* | <.01 | F(2,117)=.07 | .94 | F(2,117)=.98 | .38 | F(2,117)=.17 | .84 | F(1,117)=.21 | .65 | F(2,117)=.19 | .82 |
|  | FFAUS, 2nd half | F(1,118)=.07 | .79 | F(3,118)=1.04 | .38 | F(2,118)=1.99 | .14 | F(2,118)=.09 | .91 | F(2,118)=.05 | .95 | F(1,118)=5.24** | .02 | F(2,118)=1.03 | .36 |
|  | SFAUS | F(1,118)=.97 | .33 | F(3,118)=.78 | .51 | F(2,118)=1.08 | .34 | F(2,118)=.78 | .46 | F(2,118)=.38 | .68 | F(1,118)=1.41 | .24 | F(2,118)=1.86 | .16 |

**n*=2 in the group that differed from the others

** *n*=8 in the group that differed from the others

**Appendix F** Unadjusted means and standard deviations (*SD*) before and after the walk in Study 2, grouped by study condition

|  |  |  |  |  |  |  |  | Sustained attention to response task | | | | | | | | | | | |
| --- | --- | --- | --- | --- | --- | --- | --- | --- | --- | --- | --- | --- | --- | --- | --- | --- | --- | --- | --- |
|  |  | Restoration | | Valence | | Activation | | Commission errors | | RT | | SDRT | | FFAUS, 1st half | | FFAUS, 2nd half | | SFAUS | |
| Condition |  | Before | After | Before | After | Before | After | Before | After | Before | After | Before | After | Before | After | Before | After | Before | After |
| No tasks | Mean | 4.53 | 5.15 | 6 | 7.3 | 5.78 | 4.9 | 10.76 | 8.95 | 376.12 | 390.17 | 89.67 | 83.07 | 94.01 | 63.02 | 92.85 | 77.45 | 417.15 | 374.15 |
|  | *N* | 40 | 40 | 40 | 40 | 40 | 40 | 41 | 41 | 41 | 41 | 41 | 41 | 40 | 40 | 40 | 40 | 40 | 40 |
|  | *SD* | 0.7 | 0.72 | 1.63 | 1.24 | 1.61 | 1.75 | 5.42 | 6.43 | 71.1 | 91.86 | 32.61 | 36.46 | 107.28 | 39.02 | 76.67 | 56.51 | 394.52 | 475.72 |
| Restoration-enhancement tasks | Mean | 4.38 | 5.29 | 6 | 7.73 | 5.55 | 4.85 | 11.82 | 10.24 | 378.07 | 379.75 | 89.91 | 84.55 | 83.48 | 70.22 | 84.64 | 75.69 | 387.72 | 345.5 |
|  | *N* | 39 | 39 | 40 | 40 | 40 | 40 | 38 | 38 | 38 | 38 | 38 | 38 | 38 | 38 | 38 | 38 | 38 | 38 |
|  | *SD* | 0.87 | 0.71 | 1.68 | 0.85 | 1.4 | 1.59 | 5.03 | 5.41 | 71.65 | 88.73 | 33.79 | 33.49 | 86.78 | 51.25 | 70.39 | 61.13 | 402.76 | 346.63 |
| Awareness-enhancement tasks | Mean | 4.58 | 5.27 | 6 | 7.38 | 5.43 | 5.13 | 9.68 | 9.08 | 405.84 | 401.8 | 102.66 | 93.23 | 94.88 | 81.83 | 95.77 | 93.09 | 544.45 | 502.82 |
|  | *N* | 39 | 40 | 40 | 40 | 40 | 40 | 40 | 40 | 40 | 40 | 40 | 40 | 39 | 40 | 40 | 40 | 40 | 40 |
|  | *SD* | 1.04 | 0.91 | 2.06 | 1.25 | 1.82 | 1.77 | 5.54 | 5.86 | 86.73 | 87.5 | 34.26 | 34.74 | 74.04 | 57.74 | 59.48 | 76.37 | 477.36 | 593.46 |
| Total | Mean | 4.5 | 5.24 | 6 | 7.47 | 5.58 | 4.96 | 10.73 | 9.4 | 386.73 | 390.75 | 94.12 | 86.96 | 90.88 | 71.72 | 91.19 | 82.19 | 450.82 | 408.54 |
|  | *N* | 118 | 119 | 120 | 120 | 120 | 120 | 119 | 119 | 119 | 119 | 119 | 119 | 117 | 118 | 118 | 118 | 118 | 118 |
|  | *SD* | 0.88 | 0.78 | 1.79 | 1.14 | 1.61 | 1.7 | 5.37 | 5.91 | 77.44 | 89.1 | 33.82 | 34.95 | 89.97 | 50.11 | 68.76 | 65.21 | 428.73 | 485.35 |
